# Sleep-Disordered Breathing and Prognosis after Ischemic Stroke: It Is Not Apnea-Hypopnea Index That Matters

**DOI:** 10.3390/diagnostics13132246

**Published:** 2023-07-03

**Authors:** Lyudmila Korostovtseva, Mikhail Bochkarev, Valeria Amelina, Uliana Nikishkina, Sofia Osipenko, Anastasia Vasilieva, Vladislav Zheleznyakov, Ekaterina Zabroda, Alexey Gordeev, Maria Golovkova-Kucheryavaia, Stanislav Yanishevskiy, Yurii Sviryaev, Aleksandra Konradi

**Affiliations:** 1 Almazov National Medical Research Centre, 197341 St. Petersburg, Russiazabroda.en.1702@gmail.com (E.Z.);; 2 Department of Clinical Psychology, Herzen State Pedagogical University, 191186 St. Petersburg, Russia; 3 Medical Faculty, Pavlov University, 197022 St. Petersburg, Russia; 4 Biology Faculty, Saint Petersburg State University, 199034 St. Petersburg, Russia

**Keywords:** stroke, ischemic stroke, stroke outcome, sleep-disordered breathing, hypoxemia burden

## Abstract

Background: Sleep-disordered breathing (SDB) is highly prevalent after stroke and is considered to be a risk factor for poor post-stroke outcomes. The aim of this observational study was to evaluate the effect of nocturnal respiratory-related indices based on nocturnal respiratory polygraphy on clinical outcomes (including mortality and non-fatal events) in patients with ischemic stroke. Methods: A total of 328 consecutive patients (181 (55%) males, mean age 65.8 ± 11.2 years old) with confirmed ischemic stroke admitted to a stroke unit within 24 h after stroke onset were included in the analysis. All patients underwent standard diagnostic and treatment procedures, and sleep polygraphy was performed within the clinical routine in the first 72 h after admission. The long-term outcomes were assessed by cumulative endpoint (death of any cause, new non-fatal myocardial infarction, new non-fatal stroke/transient ischemic attack, emergency revascularization, emergency hospitalization due to the worsening of cardiovascular disease). A Cox-regression analysis was applied to evaluate the effects of nocturnal respiratory indices on survival. Results: The mean follow-up period comprised 12 months (maximal—48 months). Patients with unfavourable outcomes demonstrated a higher obstructive apnea-hypopnea index, a higher hypoxemia burden assessed as a percent of the time with SpO_2_ < 90%, a higher average desaturation drop, and a higher respiratory rate at night. Survival time was significantly lower (30.6 (26.5; 34.7) versus 37.9 (34.2; 41.6) months (Log Rank 6.857, *p* = 0.009)) in patients with higher hypoxemia burden (SpO_2_ < 90% during ≥2.1% versus <2.1% of total analyzed time). However, survival time did not differ depending on the SDB presence assessed by AHI thresholds (either ≥5 or ≥15/h). The multivariable Cox proportional hazards regression (backward stepwise analysis) model demonstrated that the parameters of hypoxemia burden were significantly associated with survival time, independent of age, stroke severity, stroke-related medical interventions, comorbidities, and laboratory tests. Conclusion: Our study demonstrates that the indices of hypoxemia burden have additional independent predictive value for long-term outcomes (mortality and non-fatal cardiovascular events) after ischemic stroke.

## 1. Introduction

Stroke is the second-leading cause of death and the biggest cause of major disability leading to a significant economic burden. Ischemic stroke comprises two thirds of all incident strokes [1]. Stroke care accounts for about three percent of total health care expenditures. Despite the development of technologies and treatment approaches, the incidence and prevalence of stroke have increased over the last three decades. 

Aside from the well-established risk factors for stroke (including hypertension, obesity, glucose metabolism impairment, smoking, air pollution) [1], sleep-disordered breathing (SDB) is considered to be a potential contributing factor. The prevalence of SDB is high in acute stroke, achieving 71% and higher [2], with a clear trend to decrease in frequency and severity in the subacute and chronic phases [2,3]. Untreated obstructive sleep apnea (OSA) is associated with a two-fold increased risk in stroke incidence after adjusting for other confounders [4,5], in particular, in younger patients. The relationship between SDB and stroke is considered complex and bidirectional [4]; SDB can increase the risk of stroke directly and indirectly via amplifying the burden of other risk factors (high blood pressure, risk of atrial fibrillation, paradoxical emboli, etc. [6]), while stroke predisposes to SDB by the disruption of the central mechanisms of respiration regulation [7]. However, the results of the studies evaluating the effect of CPAP therapy on the risk of stroke are inconclusive, with the observational studies supporting the benefits and the results of randomized clinical trials (RCT) being controversial [4]. Such a controversy might be related to a low compliance [4,8], high drop-out [9], and other factors, as well as to the heterogeneity of populations and SDB per se. The latter can differ by pathophysiological mechanisms, clinical manifestations, and, as a consequence, by the response to therapy [10]. Thus, the estimation of SDB should not be based solely on the apnea-hypopnea index (AHI), but should also include other metrics for better evaluation of SDB subtypes and the choice of management approach [11,12,13,14]. Among potential measures, hypoxemia is a candidate, and the potential benefits and harms of supplemental oxygen therapy in intensive care settings has been a question of debate (currently, supplemental oxygen is not recommended in non-hypoxemic subjects) [15,16]. In recent years, several studies have shown that SDB-related hypoxia burden is associated with cardiovascular diseases [13] and negative prognosis [17]. Azarbarzin et al. (2019) evaluated the impact of the hypoxic burden (estimated as the total area under the respiratory event-related desaturation curve) on mortality in a large cohort combined from the Osteoporotic Fractures in Men Study (MrOS) Sleep Study and the Sleep Heart Health Study (SHHS). They found that the measure of hypoxic burden was independently associated with cardiovascular mortality, while the conventional AHI metric did not show such an association.

In a large, multicentre cohort (*n* = 3597), Blanchard et al. (2021) reported a higher risk of incident stroke associated with higher hypoxic burden assessed by the PSG-derived indices (adjusted hazard ration (HR) 1.30, 95% CI 1.05–1.61, *p* = 0.02) [18]. Being a strong arrhythmogenic factor, hypoxia might contribute to atrial fibrillation and predispose to cerebrovascular events [19,20]. SDB-related nocturnal hypoxemia is independently associated with brain white matter hyperintensities, which are presumed to have vascular origin [21]. 

In stroke patients, hypoxia is observed in about 2/3 of patients within the first 1–3 days after stroke onset, and about 25% of stroke patients who present as normoxic at admission develop nocturnal hypoxia [22]. In stroke, additional factors may affect the hypoxemia burden, including the disrupted central regulation of respiration, weakness of the respiratory muscles, dysphagia, and aspiration-related complications including bronchopulmonary infections, etc. Stroke volume was also shown to correlate with nocturnal hypoxemia [23]. However, studies assessing the impact of the hypoxemia burden on prognosis in stroke survivors are limited. 

We hypothesized that the hypoxemia burden and other nocturnal respiratory-related indices, rather than AHI, are associated with outcomes after ischemic stroke.

The aim of this observational study was to evaluate the effect of nocturnal respiratory-related indices based on nocturnal respiratory polygraphy on clinical outcomes (including mortality and non-fatal events) in patients with ischemic stroke. 

## 2. Materials and Methods

### 2.1. Study Design

The study was designed as an open, observational, cohort study. The study was conducted in accordance with the Declaration of Helsinki, and the study protocol was approved by the Local Ethical Committee of Almazov National Medical Research Centre (protocol №1612-21-02). All patients signed informed consent before inclusion. The current analysis is performed as a part of the study registered at ClinicalTrials.gov Identifier: NCT05242393. 

Among patients admitted to the Almazov National Medical Research Centre’s Stroke Unit in the period 2018–2023, we selected those eligible according to the inclusion/exclusion criteria.

Inclusion criteria: admission to the Stroke Unit within 24 h after symptom onset, age 18–80 years, ischemic stroke verified by clinical and neuroimaging examination (MRI or CT scans) caused by the occlusion of the anterior, middle, or posterior cerebral artery and their branches, signed informed consent.

Exclusion criteria: loss of consciousness, primary hemorrhagic stroke, need for intubation or O_2_ supply >2 L/min, clinically unstable or life threatening condition, congestive heart failure with reduced ejection fraction (≤45%) or New York Heart Association (NYHA) classification III-IV functional class, known psychiatric diseases, known progressive neurological diseases, concomitant benzodiazepine medication, drug or alcohol abuse, pregnancy, disability to participate in the study, non-valid sleep study, CPAP treatment before stroke, or initiation of CPAP treatment after stroke onset.

### 2.2. Study Cohort

**Figure 1:**
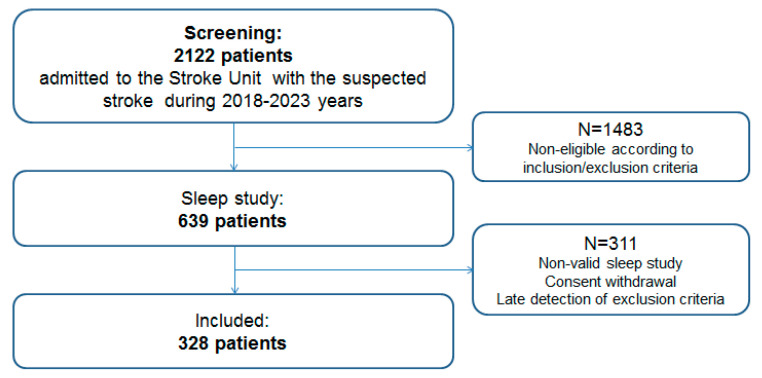
Flowchart of the cohort selection.

Altogether 2122 patients consecutively admitted with suspected stroke were screened during the period 2018–2023 (average age 65.7 ± 13.6 years, males 1036 (49%), NIHSS Me (Min Max) 3 (0:32) score). According to the inclusion/exclusion criteria, 639 were considered eligible and underwent a sleep study. Due to non-valid sleep study results, informed consent withdrawal, early drop-out, or late detection of exclusion criteria, 311 patients were excluded. Thus, in total, 328 consecutive patients were included in the analysis (Figure 1). 

### 2.3. Procedures

Upon admission, all patients underwent standard routine diagnostic procedures and received standardized treatment according to the guidelines for acute stroke management. 

The neurological status and its related dynamics in the acute stroke period were assessed within the clinical routine by the National Health Institute Stroke Scale (NIHSS) at admission and at discharge (scored from 0 to 42, with higher values indicating more severe stroke). The severity of stroke was classified as follows: 0—no stroke, 1–4—minor stroke, 5–15—moderate stroke, 16–42—severe stroke [8]. Functional state and outcomes were assessed at admission and at discharge using the following tools: activity Barthel index (scored from 0 to 100, with higher values indicating higher performance of daily activities [24]), Rivermead mobility index [25] (scored from 0 to 15, with higher values indicating higher functional mobility), and modified Rankin scale [26] (mRs, scored from 0 to 6, with lower values indicating lower disability and a score of 6 indicating death).

Information on medical history for comorbidities preceding therapy was collected within clinical routine examination and obtained from medical records. Neuroimaging studies (CT and/or MRI) for diagnosis verification were performed in all patients within clinical routine at admission. The TOAST (Trial of Org 10172 in Acute Stroke Treatment) classification was used to define the etiological subtypes of ischemic stroke as follows: (1) large-artery atherosclerosis, (2) cardioembolism, (3) small-vessel occlusion (lacunar stroke), (4) other determined etiology, (5) undertermined etiology [27]. 

Blood samples were collected within clinical routine at admission, and the following parameters were assessed: complete blood count, biochemical tests including serum lipids, creatinine (estimated glomerular filtration rate calculated using CKD-EPI formula), glucose, C-reactive protein, and fibrinogen. 

A sleep study was performed within the clinical routine within the first 72 h after admission and included the registration of the following parameters: oronasal airflow, pulseoximeter, abdominal and thoracic movements by inductive plethysmography, and body position. The scoring of sleep-related events was performed manually by two trained specialists according to the AASM 2.4 scoring rules [28]. The following parameters were assessed: apnea-hypopnea index (AHI, episodes/h), obstructive (OAH), central (CAH) and mixed (MAH) episodes index, oxygen desaturation index (ODI, episodes/h), average and minimal SpO_2_ (%), time percent spent with SpO_2_ < 90% and <85% (percent of total analyzed time), average desaturation drop (%), respiratory rate (per minute), time spent in supine/non-supine position (percent of total analyzed time), supine AHI (episodes/h), and average duration of apnea-hypopnea episodes (sec). Two SDB diagnostic thresholds were evaluated—AHI ≥ 5/h and AHI ≥ 15/h. Based on the AHI index the following severity categories were distinguished: mild SDB (AHI 5–14.9/h), moderate SDB (AHI 15–29.9/h), and severe SDB (AHI ≥ 30/h).

### 2.4. Endpoints and Follow-Up

The information about endpoints was collected during follow-up visits scheduled at 3–6 months, and annually afterwards. All patients were invited for outpatient face-to-face visits; however, in case of non-attendance, the data were collected by phone. Non-scheduled visits were performed when necessary. 

In case of failure to contact, the most recent known data were used. The follow-up period was assessed as the time from inclusion date to the date of the last visit and/or the date of the last known information (obtained from patients’ relatives/caregivers or physicians, from medical records, i.e., dates of cardiovascular events). 

The cumulative endpoint included: death by any cause, new non-fatal myocardial infarction (MI), new non-fatal stroke/transient ischemic attack (TIA), emergency revascularization, or emergency hospitalization due to the worsening of cardiovascular disease. 

### 2.5. Statistical Analysis

Statistical analysis was carried out with the use of IBM SPSS Statistics v.26.0. No imputation of missing values was performed. 

The distribution of continuous variables was assessed by a Kolmogorov–Smirnov test. Continuous variables are shown as mean (M) ± standard deviation (SD) or as median (Me) and minimal and maximal values (Min; Max), depending on the distribution type. Nominal variables are presented as frequencies and/or as percentages. A Chi-square test and Fisher’s exact test were used to compare categorical variables in two independent groups, and a McNemar test was used to compare frequencies in related samples. Continuous variables were compared by Mann–Whitney (2 groups) or Kruskall–Wallis (>2 groups) tests. An ANOVA or Wilcoxon test were used for the related samples. 

Correlation Spearman analysis was used to evaluate the associations between variables. A Kaplan–Meier analysis with the assessment of Log Rank criteria was applied to evaluate survival. For predictors assessment, a Cox-regression analysis with backward stepwise selection was applied. The occurrence of cumulative endpoint was included as a dependent variable. As potential independent variables, we included the following factors based on the common understanding of their potential association with the outcome and the results of the univariate regression analysis (variables considered important at *p*-level < 0.2): age (continuous), sex (m/f), stroke subtype according to TOAST classification (categorical), NIHSS score at discharge (continuous), thrombolytic therapy at admission (yes/no), emergency stroke-related vascular intervention at admission (yes/no), presence of atrial fibrillation (yes/no), previous stroke/TIA (yes/no), coronary artery diseases (CAD) (yes/no), obesity (body mass index > 30 kg/m^2^, yes/no), diabetes mellitus (yes/no), hypertensive crisis at stroke onset (yes/no), serum levels of triglycerides (continuous), C-reactive protein (continuous), platelet count (continuous), eGFR (continuous), and nocturnal respiratory rate (yes/no, cut-off at 15.6/min). Among sleep-related parameters, we included (not simultaneously due to their high collinearity) the following parameters: time spent at SpO_2_ < 90% (yes/no, cut-off at 2.1% of total analyzed time), AHI, obstructive OAH, and average desaturation drop (yes/no, cut-off at 3.65%). The variables showing high collinearity were not included simultaneously in the model; in this case, the choice was based on clinical understanding of the higher significance of the parameter. An ROC-curve analysis was used to select the optimal cut-off values of the studied parameters. 

According to the pre-defined calculation, the planned cohort sample size is ~384 patients. This report includes preliminary results of the study.

All statistical tests were two-sided and conducted at the 5% significance level. The rounding was up to the hundredth; in case of borderline values, they were rounded up to the thousandth. 

## 3. Results

### 3.1. Patient Characteristics

In total, 328 patients (181 males, 55%) were included in the analysis. Mean age was 65.8±11.2 years old (Table 1). The majority of patients (80%) were hypertensives, with 10.6% presenting a hypertensive crisis at stroke onset. Half of the patients had other cardiovascular comorbidities, including CAD (42.1%), heart rhythm disorders (25%), valvular disease (10%), and others. Twenty-eight percent of patients had stroke/TIA in past.

The mean NIHSS at admission was 4 (1; 25) score. Mild stroke was diagnosed in 41.4% patients, moderate—in 32%, severe—in 9%. According to TOAST classification, in the majority of cases (37.8%), the etiology remained undetermined; cardioembolic strokes comprised 31.4%; atherothrombotic 18.6%; small-vessel disease 10%; and other etiology was verified in 1.5% of cases. Middle cerebral artery was the most frequently affected (75.3%), while in 8.5% of cases, the involvement of several arteries was verified (Table 2). 

All patients received standard care according to the guidelines for the management of ischemic stroke, including anticoagulants, antiagregants, hypolipidemic, antihypertensive medications, and other treatments if needed. Forty-three (13.1%) patients received thrombolytic therapy at stroke onset. In the majority of cases, thrombolytic therapy was not indicated due to the referral/admission outside the therapeutic window or small stroke. Emergency stroke-related vascular intervention (thromboextraction/thromboaspiration) was performed in each fifth patient (Table 2).

### 3.2. Follow-Up and Outcomes

The mean follow-up period comprised 12 months, while maximal follow-up was 48 months. At one-year follow-up, the dropout rate was 52% (172 patients).

In total, 65 (19.8%) endpoints were registered; among them, 32 (9.8%) lethal cases, 18 (5.5%) recurrent strokes, 2 (0.6%) recurrent TIAs, 5 (1.5%) MIs, and 14 (4.3%) emergency hospitalizations (8 due to stroke/TIA and MI and 6 due to other cardiovascular reasons).

Patients who achieved endpoints were older and had higher rates of comorbidities. Thus, among them, the rates of hypertension, obesity, CAD and previous MI, atrial fibrillation (AF), and previous pulmonary thromboembolism were higher. However, the frequency of previous cerebrovascular events was similar in those who achieved endpoints and those with event-free survival (Table 1). At admission, patients who achieved endpoints during follow-up demonstrated higher inflammatory response, had lower levels of triglycerides, and slightly lower eGFR (Table 3). 

Patients with unfavourable long-term outcomes had higher NIHSS scores at admission, higher rates of moderate-severe stroke, and poorer functional scores at discharge (Barthel index, Rivermead index, mRS) (Table 4).

### 3.3. Sleep-Disordered Breathing in the Studied Cohort

Based on the sleep study at baseline, the mean AHI comprised 15.4 (0.0; 87.8) episodes/h. Although SDB (AHI ≥ 5/h) was more frequently found in the group with achieved endpoints (50 patients (76.9%)) compared to the event-free survival group (170 patients (64.6%)), the difference was not significant (χ^2^ = 3.561, *p* = 0.076). Those with unfavourable outcomes demonstrated higher obstructive OAH, hypoxemia burden assessed as percent of time with SpO_2_ < 90%, higher average desaturation drop, and higher respiratory rate at night. They also spent more time in the supine position during the sleep study and had a higher hypopnea index while lying on their backs (Table 5). 

Based on the ROC-curve analysis, the cut-off values for the respiratory parameters showing significant difference in the groups were detected. The OAH cut-off value was 3.5/h with sensitivity 60%, specificity 54% (AUC 0.585 95% CI (0.506–0.664), *p* = 0.040), percent of total analyzed time with SpO_2_ < 90%: 2.1% with sensitivity 61%, specificity 46% (AUC 0.609 95% CI (0.531–0.687), *p* = 0.006), respiratory rate at night: 15.6 breaths/min with sensitivity 71%, specificity 46% (AUC 0.602 95% CI (0.516–0.688), *p* = 0.020), and average desaturation drop: 3.65% with sensitivity 78%, specificity 56% (AUC 0.600 95% CI (0.518–0.681), *p* = 0.016). 

### 3.4. Survival

**Figure 2:**
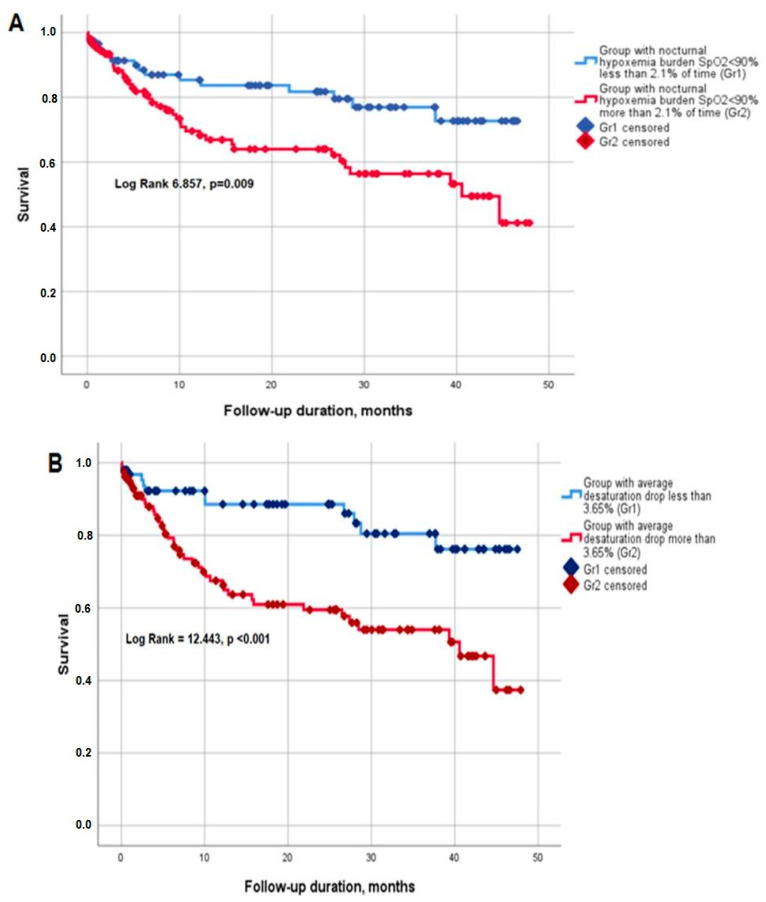
Survival (Kaplan–Meier curves) depending on the hypoxemia burden: (**A**) nocturnal hypoxemia SpO_2_ < 90%, (**B**) average desaturation drop.

The mean survival time was 33.8 (95%CI 31.0; 36.7) months. When survival was assessed depending on the nocturnal hypoxemia burden (SpO_2_ < 90% during ≥2.1% of total analyzed time), the mean survival time in the group with a higher hypoxemia burden ≥2.1% was 30.6 (26.5; 34.7) months versus 37.9 (34.2; 41.6) months in the group with a lower hypoxemia burden (<2.1%) (Log Rank 6.857, *p* = 0.009) (Figure 2A). Similarly, event-free survival time was significantly shorter in patients with higher desaturation drops (≥3.65%) during the night (29.3 (25.2; 33.3) versus 40.5 (36.9; 44.2) months in subgroups with an average nocturnal desaturation drop ≥ 3.65% and <3.65%; Log Rank 12.443, *p* < 0.001) (Figure 2B).

**Figure 3:**
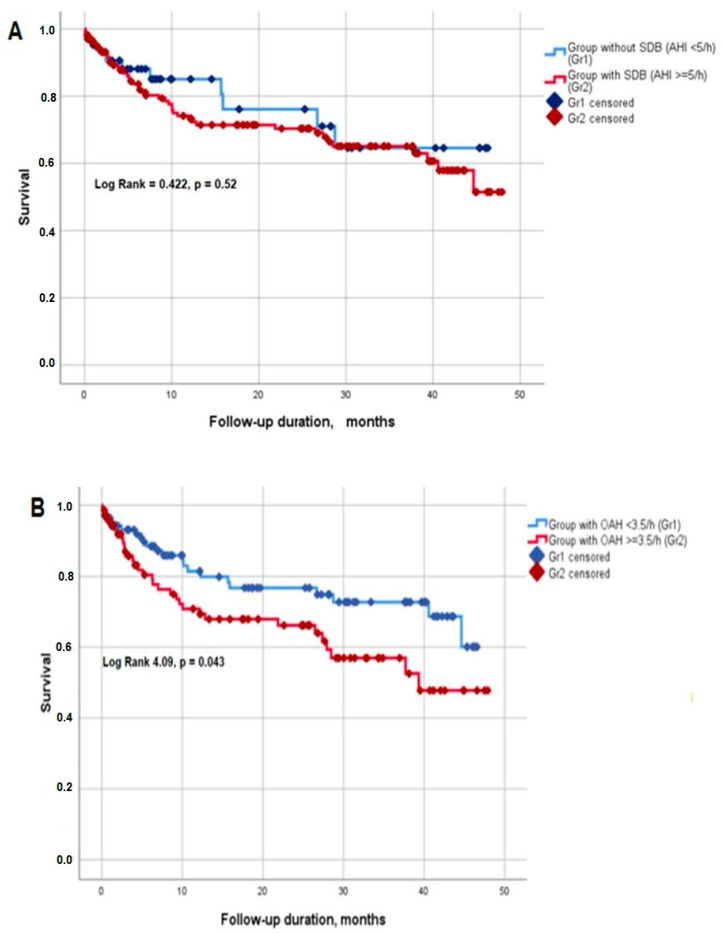
Survival (Kaplan–Meier curves) depending on the presence of sleep-disordered breathing: (**A**) AHI < 5 versus AHI ≥ 5/h, (**B**) OAH < 3.5 versus OAH ≥ 3.5/h.

When survival rate was assessed depending on the SDB severity parameters, the mean survival time was slightly longer in patients with OAH over 3.5/h (36.2 (32.5; 39.9) versus 31.0 (26.5; 35.4) months, *p* = 0.043), while it did not differ in patients with and without SDB (AHI < 5 versus AHI ≥ 5/h, *p* = 0.52) (Figure 3A,B). Similar results were obtained when the threshold of AHI ≥ 15/h was applied (*p* = 0.69).

The multivariable Cox proportional hazards regression (backward stepwise analysis) model demonstrated that the parameters of hypoxemia burden were significantly associated with survival time (Table 6), independent of age, stroke severity, stroke-related medical interventions, comorbidities, and laboratory tests. We repeated the analysis using forward stepwise analysis and obtained similar results. 

We performed a subanalysis in a subgroup of patients with previous stroke (*n* = 93) (Supplemental Material). Although the survival rate was poorer in those with a higher nocturnal hypoxemia burden [29.2 (21.9; 36.4) months] compared to those with a lower nocturnal hypoxemia burden [38.0 (33.8; 42.1)] (Log Rank 5.366, *p* = 0.021) (Figure S1), in a multivariate Cox regression analysis, age was the only factor associated with survival time.

## 4. Discussion

In a cohort observational study, we found that nocturnal hypoxemia burden is significantly associated with an unfavourable prognosis and lower event-free survival time in patients with ischemic stroke, independent of age, sex, stroke severity, stroke-related medical interventions (thrombolytic therapy and emergency stroke-related vascular intervention), comorbidities (CAD, AF, obesity, diabetes mellitus, previous stroke/TIA), and laboratory tests (including, inflammatory response, platelet count and triglyceride level). In addition, we found that regarding SDB conventional characteristics, only obstructive AHI was slightly associated with event-free survival after ischemic stroke, while the presence of SDB and total AHI did not demonstrate an association with long-term outcomes.

Our results are concordant with the recently developed position regarding the limited diagnostic and prognostic value of the AHI as a single metric of SDB severity [10,11,29]. In the 1970s, some authors had already expressed doubts about appropriateness of AHI as a diagnostic SDB measure. The SDB-related cardiovascular consequences are determined by a number of systemic effects, including hypoxemia, sympathetic overactivity, blood pressure, and intrathoracic pressure swings [10], which are not reflected by a single AHI metric. 

In a meta-analysis evaluating the effect of OSA on neuropsychological functioning, Dean W. Beebe et al. (2003) emphasized that the differentiation of patients solely based on AHI and the acceptance of AHI as the main severity metric might be misleading, while hypoxemia could be a more important factor, in particular, for neurocognitive functioning [30]. This corresponds to the observations of Good et al. (1996), who showed that short- and long-term functional outcomes in post-stroke survivors (assessed by Barthel Index at discharge and 12-month follow-up) are associated with the oximetry measures – mean SpO_2_ saturation, desaturation index, and percentage of recording time spent at SpO_2_ < 90% [31]. In a recent analysis by Xu et al. (2023), severe OSA associated with high ODI and reduced slow-wave sleep was predictive of poor functional outcome [32]. Good et al. (1996) suggested that tissue hypoxia limits the neuroplasticity and contributes to the widening of penumbra. In the setting of intermittent hypoxemia observed in SDB, an adaptive antihypoxic response cannot be appropriately developed. 

The higher predictive significance of other metrics beyond AHI might explain the contradictory results of prospective observational and RCT assessing long-term prognosis after stroke [33,34,35], in addition to such factors as high drop-out and low compliance [8] to CPAP [4,9], and might be responsible for the multidirectional effects of SDB. In animal stroke models, intermittent hypoxia leads to more severe brain damage and poorer sensorimotor performance, which correlates with the dysregulation of autonomic response, increases in blood pressure, and disrupted sleep patterns [34].

In patients after ischemic stroke, a number of additional factors can contribute to the higher heterogeneity of SDB and potential different SDB-related long-term consequences. It was shown that the prevalence and type of SDB is greatly correlated with the subtype, location, etiology, and phase of stroke [2,36]. In addition, although OSA is the most prevalent SDB type in acute stroke, central SDB are also rather common, in particular, in the acute phase. The clinical manifestations of SDB can be fuzzy in patients after stroke and can be masked by the neurologic deficit, which also explains the low accuracy of the existing pre-screening tools [37,38]. Schutz et al. (2019) detected three distinct clinical phenotypes of SDB in post-stroke patients, including severe strokes, younger patients with mild strokes and relatively mild OSA, and severe OSA with high prevalence of co-morbidities. Future studies of the evaluation of the prognostic significance of these variants are needed. In the present analysis, we did not evaluate clinical manifestations of SDB and their association with the long-term outcomes, as we mainly focused on instrumental data obtained by a sleep study in the acute phase of stroke (within 48-72 h after admission to a stroke unit), when taking medical history and filling in screening questionnaires are associated with certain difficulties. Moreover, as mentioned before, the existing pre-screening tools demonstrate low specificity [37], while the common SDB-related symptoms might be blurred by neurologic deficit, sleep disruption in the hospital setting, emotional disturbances, etc.

In our analysis, several other factors besides hypoxemia burden appeared to be associated with poor long-term prognosis, including age, neurological deficit (NIHSS at discharge), stroke etiology subtype (borderline significant), diabetes mellitus, atrial fibrillation, and several laboratory parameters (C-reactive protein, platelet count, and triglyceride level). For all these factors, the association with stroke outcomes is well established; thus, our data provides evidence on the prognostic role of hypoxemia burden in addition to the common risk factors in post-stroke. A recent study showed that atrial fibrillation as a cause for cardioembolic stroke is the most prevalent among patients with sleep apnea; however, the multivariate analysis did not confirm association and indicated only age as the major predictor. The authors used AHI≥15/h as the cut-off and diagnostic measure of sleep apnea and did not consider other sleep-related respiratory indices, which might have affected the results [39]. Interestingly, in our cohort, low triglyceride level was associated with poorer outcome. Despite the generally accepted concept of the deleterious cardiovascular effects of high triglyceride levels [40,41], a “triglyceride paradox” has been reported in stroke survivors [42,43,44]. Such association can be explained by the J-shaped curve phenomenon with regard to triglyceride levels and their impact on prognosis confirmed in some studies [45,46]. Other authors attribute this association to the neuroprotective properties of triglycerides [44], as well as a poor nutritional status prior to stroke, and consider low triglyceride level as a marker of nutrition deficiency [42,43]. 

There are certain limitations that should be considered when interpreting our study results. First, our study cohort had some characteristics that may have caused selection bias, e.g., the NIHSS score at baseline was comparably low, and no patients with very severe stroke (NIHSS > 25 score) were included. Additionally, we excluded patients with severe heart failure, which might have affected the prevalence of SDB and its subtypes in our cohort. Therefore, our results cannot be generalized to all stroke patients. However, our cohort includes patients with strokes of various etiology (subtypes according to TOAST classification), different comorbidities, and who received standard care including emergency stroke-related interventions. In general, our cohort (with regard to the age, stroke severity, TOAST subtype of stroke, and other characteristics) is similar to other cohorts of patients with stroke evaluated for SDB effects on stroke outcomes [35,38]. At the same time, we applied adjustments for a number of potential important confounders, including stroke characteristics, demographical data, comorbidities, laboratory tests, and stroke-related interventions, and the role of nocturnal respiratory indices (nocturnal hypoxemia burden) remained a significant predictor of poor outcome. Secondly, we used respiratory polygraphy for SDB assessment, which could underestimate SDB severity. To overcome this limitation, we applied two cut-off values of the AHI to detect SDB (at 5 and 15 episodes/h); however, none of the approaches showed a significant association between SDB presence and survival. We did not exclude patients with predominant central sleep apnea; however, we analyzed obstructive and sleep AHI separately, and found that only obstructive AHI is slightly associated with poorer prognosis. Last but not least, the dropout rate was rather high at 12 months (52%); however, this is expected in the cohort of patients with stroke. 

## 5. Conclusions

Our study demonstrates that the indices of hypoxemia burden (percentage of time spent at SpO_2_ < 90% at night and average desaturation drop) have additional predictive value for the long-term outcomes (regarding mortality and non-fatal cardiovascular events) after ischemic stroke, independent of demographic and stroke characteristics, comorbidities, and blood test abnormalities. Future research might focus on the search for and development of an integrated parameter for prognostic modeling that includes both indices derived from sleep polygraphy and clinical characteristics. Future prospective studies are also needed to evaluate whether CPAP and other modes of positive airway pressure therapy are more beneficial in patients with a higher hypoxemia burden.

## Figures and Tables

**Table 1:** Cohort characteristics: demographics and comorbidities.

Parameter	Total (*n* = 328)	Patients with Poor Survival (*n* = 65)	Patients Free of Events (*n* = 263)	*p*-Level
Sex (m), *n* (%)	181 (55.2%)	40 (61.5%)	141 (53.6%)	χ^2^ = 1.043, *p* = 0.31
Age, years	65.8 ± 11.2	69.5 ± 9.5	64.7 ± 11.5	*p* = 0.005
HTN, *n* (%)	262 (79.8%)	64 (98.5%)	198 (75.3%)	χ^2^ = 5.700, *p* = 0.017
HTN crisis, *n* (%)	35 (10.7%)	5 (7.7%)	30 (11.4%)	χ^2^ = 1.667, *p* = 0.20
AF, *n* (%)	82 (25%)	33 (50.8%)	49 (18.6%)	χ^2^ = 19.880, *p*<0.001
Pacemaker, *n* (%)	4 (1.2%)	1 (1.5%)	3 (1.1%)	χ^2^ = 0.144, *p* = 0.70
CAD, *n* (%)	138 (42.1%)	42 (64.6%)	96 (36.5%)	χ^2^ = 8.861, *p* = 0.003
Previous MI, *n* (%)	57 (17.4%)	19 (29.2%)	38 (14.5%)	χ^2^ = 4.455, *p* = 0.035
CAD-related heart surgery, *n* (%)	28 (8.5%)	8 (12.3%)	20 (7.6%)	χ^2^ = 0.576, *p* = 0.45
Valvular disease, *n* (%)	33 (10.1%)	8 (12.3%)	25 (9.5%)	χ^2^ = 0.041, *p* = 0.84
Valvular surgery, *n* (%)	4 (1.2%)	0	4 (1.9%)	χ^2^ = 1.202, *p* = 0.27
Pulmonary thromboembolism, *n* (%)	4 (1.2%)	3 (4.6%)	1 (0.4%)	χ^2^ = 6.257, *p* = 0.012
Pulmonary hypertension, *n* (%)	17 (5.2%)	3 (4.6%)	14 (5.3%)	χ^2^ < 0.001, *p* = 0.99
Other CVD (myxoma, foramen ovale)	8 (2.4%)	0	8 (3%)	χ^2^ = 2.127, *p* = 0.15
Diabetes mellitus, *n* (%)	70 (21.3%)	18 (27.7%)	52 (19.8%)	χ^2^ = 0.432, *p* = 0.51
Dyslipidemia, *n* (%)	157 (47.9%)	35 (53.8%)	122 (46.4%)	χ^2^ = 0.065, *p* = 0.80
Obesity, *n* (%)	70 (21.3%)	22 (33.8%)	48 (18.3%)	χ^2^ = 3.889, *p* = 0.049
CKD, *n* (%)	24 (7.3%)	1 (1.5%)	23 (8.7%)	χ^2^ = 3.378, *p* = 0.066
COPD, *n* (%)	23 (7.0%)	5 (7.7%)	18 (6.8%)	χ^2^ = 0.018, *p* = 0.89
Bronchial asthma, *n* (%)	5 (1.5%)	0	5 (1.9%)	χ^2^ = 1.267, *p* = 0.26
Previous stroke/TIA, *n* (%)	93 (28.4%)	21 (32.3%)	72 (27.3%)	χ^2^ = 0.006, *p* = 0.94
PAD, *n* (%)	21 (6.4%)	7 (10.8%)	14 (5.3%)	χ^2^ = 3.404, *p* = 0.065
Thyroid disease, *n* (%)	22 (6.7%)	3 (4.6%)	19 (7.2%)	χ^2^ = 0.269, *p* = 0.60
Cancer, *n* (%)	16 (4.9%)	2 (3.1%)	14 (5.3%)	χ^2^ = 0.386, *p* = 0.53
Varicose vein disease, *n* (%)	31 (9.5%)	6 (9.2%)	25 (9.5%)	χ^2^ = 0.34, *p* = 0.85

HTN—hypertension, AF—atrial fibrillation, CAD—coronary artery disease, MI—myocardial infarction, CVD—cardiovascular disease, CKD—chronic kidney disease, COPD—chronic obstructive pulmonary disease, TIA—transient ischemic attack, PAD—peripheral artery disease.

**Table 2:** Stroke characteristics.

Parameter	Total (*n* = 328)	Patients with Poor Survival (*n* = 65)	Patients Free of Events (*n* = 263)	*p*-Level
TOAST classification				*p* = 0.023
Atherothrombotic	61 (18.6%)	13 (20%)	48 (18.3%)	
Cardioembolic	103 (31.4%)	30 (46.2%)	73 (27.8%)	
Small-vessel disease	33 (10%)	2 (3.1%)	31 (11.8%)	
Other determined etiology	5 (1.5%)	1 (1.5%)	4 (1.5%)	
Undetermined etiology	128 (37.8%)	19 (28.8%)	109 (41.4%)	
Affected cerebral artery				*p* = 0.55
Vertebrobasilar	52 (15.9%)	9 (13.8%)	43 (16.3%)	
Anterior cerebral artery	3 (0.9%)	0	3 (1.1%)	
Median cerebral artery	247 (75.3%)	49 (75.4%)	198 (75.3%)	
Several arteries	28 (8.5%)	7 (10.8%)	21 (8.0%)	
Medical interventions				
Thrombolytic therapy, *n* (%)	43 (13.1%)	7 (10.8%)	36 (13.7%)	χ^2^ = 0.39, *p* = 0.53
Emergency stroke-related vascular surgery	66 (20.1%)	14 (21.5%)	52 (19.8%)	χ^2^ = 0.101, *p* = 0.75

**Table 3:** Blood tests in patients with poor survival and with event-free survival.

Parameter	Total (*n* = 328)	Patients with Poor Survival (*n* = 65)	Patients Free of Events (*n* = 263)	*p*-Level
Glucose, mmol/L	6.29 (2.79; 33.33)	6.37 (2.79; 16.03)	6.22 (3.68; 33.33)	0.99
Total cholesterol, mmol/L	4.79 (1.97; 9.27)	4.65 (1.97; 7.64)	4.80 (2.20; 9.27)	0.63
Triglycerides, mmol/L	1.39 (0.53; 13.44)	1.27 (0.53; 3.28)	1.40 (0.59; 13.44)	0.011
HDL Cholesterol, mmol/L	1.08 (0.25; 3.43)	1.09 (0.25; 3.24)	1.08 (0.52; 3.43)	0.94
LDL Cholesterol, mmol/L	2.89 (0.52; 7.62)	2.81 (1.19; 5.86)	2.9 (0.52; 7.62)	0.99
Fibrinogen, mg/L	3.6 (0.87; 12.7)	3.9 (0.99; 12.70)	3.6 (0.87; 6.60)	0.012
C-reactive protein, mg/mL	3.57 (0.02; 138.32)	5.69 (0.02; 138.32)	3.2 (0.18; 130.62)	0.011
Hemoglobin, g/L	143.2 (69.9; 197.5)	142.5 (81.7; 171.2)	143.3 (69.9; 197.5)	0.52
Creatinine, mcmol/L	80.7 (38; 222.4)	81.0 (44.0; 129.0)	80.3 (38.0; 222.4)	0.61
eGFR, ml/min/1.73 m^2^	85.6 (51.2; 140.0)	83.1 (64.5; 104.6)	86.2 (51.2; 140.0)	0.046
Red blood cell count, *10^12^/L	4.76 (2.70; 6.21)	4.7 (3.5; 6.0)	4.8 (2.7; 6.2)	0.79
White blood cell count, *10^9^/L	8.1 (2.4; 20.8)	8.8 (3.4; 18.3)	7.9 (2.4; 20.8)	0.13
Hematocrit, %	42.7 (30; 109)	42.5 (30; 52.5)	42.8 (30; 109)	0.82
Platelets, *10^9^/L	218 (67; 506)	228 (67; 386)	213 (48; 506)	0.14

HDL—high-density lipoproteins, LDL—low-density lipoproteins, eGFR—estimated glomerular filtration rate.

**Table 4:** Neurological status and functional state at admission and at discharge in patients with poor survival and with event-free survival.

Parameter	Total (*n* = 328)	Patients with Poor Survival (*n* = 65)	Patients Free of Events (*n* = 263)	*p*-Level
NIHSS baseline, score	4 (1; 25)	6 (1; 25)	4 (1; 25)	0.025
NIHSS baseline ≥ 5 scores, *n* (%)	135 (41.2%)	38 (58.5%)	97 (36.9%)	χ^2^ = 4.904, *p* = 0.030
NIHSS discharge, score	3 (0; 23)	3(0; 23)	3 (0; 19)	0.083
Barthel index baseline, score	60 (0; 100)	50 (0; 100)	60 (0; 100)	0.132
Barthel index discharge, score	90 (0; 100)	80 (0; 100)	90 (0; 100)	0.018
mRS baseline, score	3 (0; 5)	4 (0; 5)	3 (1; 5)	0.124
mRS discharge, score	3 (0; 6)	3 (0; 6)	2 (0; 5)	0.005
Rivermead index baseline, score	3.5 (0; 15)	3 (0; 15)	4 (0; 15)	0.105
Rivermead index on the 2nd day (at neurology department), score	5 (0; 15)	3.5 (0; 15)	5 (0; 15)	0.045
Rivermead index discharge, score	12 (0; 15)	8 (0; 15)	12 (0; 15)	0.019

**Table 5:** Respiratory parameters in patients with poor survival and with event-free survival.

Parameter	Total (*n* = 328)	Patients with Poor Survival (*n* = 65)	Patients Free of Events (*n* = 263)	*p*-Level
AHI, episodes/h	15.4 (0.0; 87.8)	17.2 (0.6; 87.8)	14.6 (0.0; 87.8)	0.42
AHI supine, episodes/h	19.4 (0.0; 86.9)	14.0 (0.0; 86.2)	20.1 (0.0; 86.9)	0.39
HI, episodes/h	5.9 (0.0; 51.2)	5.0 (0.0; 38.0)	6.1 (0.0; 51.2)	0.39
HI supine, episodes/h	7.0 (0.0; 47.8)	4.7 (0.0; 30.7)	7.8 (0.0; 47.8)	0.008
ODI, episodes/h	12.0 (0.0; 80.9)	13.7 (0.0; 80.4)	12.0 (0.0; 80.9)	0.57
AI, episodes/h	5.6 (0.0; 82.9)	7.1 (0.0; 52.8)	4.7 (0.0; 82.9)	0.12
OAH, episodes/h	3.3 (0.0; 56.7)	4.8 (0.0; 50.6)	2.9 (0.0; 56.7)	0.038
CAH, episodes/h	0.4 (0.0; 68.3)	0.2 (0.0; 26.9)	0.4 (0.0; 68.3)	0.17
MAH, episodes/h	0.1 (0.0; 31.9)	0.1 (0.0; 19.6)	0.1 (0.0; 31.9)	0.44
Respiratory rate at night, breaths/min	16.1 (10.6; 29.0)	17.0 (11.3; 27.8)	15.9 (9.1; 29.0)	0.018
Average SpO_2_, %	92.9 (78.7; 97.8)	92.7 (78.7; 97.7)	92.9 (83.0; 97.8)	0.45
Minimal SpO_2_, %	81.0 (51.0; 95.0)	80.0 (52.0; 93.0)	81.0 (51.0; 95.0)	0.12
Average desaturation drop, %	3.9 (1.3; 9.4)	4.1 (1.3; 7.1)	3.8 (2.5; 9.4)	0.023
SpO_2_ < 90%, percent of total analyzed time	3.7 (0.0; 87.9)	6.2 (0.0; 48.3)	2.9 (0.0; 87.9)	0.018
SpO_2_ < 85%, percent of total analyzed time	0.1 (0.0; 64.0)	0.35 (0.0; 23.2)	0.1 (0.0; 64.0)	0.033
Time in the supine position, percent of total analyzed time	65.8 (0.0; 100.0)	84.8 (0.0; 100.0)	64.2 (0.0; 100.0)	0.026
Time in the non-supine position, percent of total analyzed time	30.1 (0.0; 100.0)	11.6 (0.0; 100.0)	34.6 (0.0; 100.0)	0.029
SDB severity				*p* = 0.074
No SDB, *n* (%)	108 (32.9%)	15 (23.1%)	93 (35.4%)	
Mild, *n* (%)	71 (21.6%)	16 (24.6%)	55 (20.9%)	
Moderate, *n* (%)	60 (18.3%)	12 (18.5%)	48 (18.3%)	
Severe, *n* (%)	89 (27.1%)	22 (33.8%)	67 (25.5%)	
Tachypnoe at night ≥ 15.6/min, *n* (%)	160 (48.8%)	40 (61.5%)	120 (45.6%)	χ^2^ = 3.837, *p* = 0.050
Hypoxemia (SpO_2_ < 90%) ≥2.1% of total analyzed time, *n* (%)	165 (50.3%)	42 (64.6%)	123 (46.8%)	χ^2^ = 5.002, *p* = 0.025
Average desaturation drop ≥ 3.65%, *n* (%)	163 (49.7%)	45 (69.2%)	118 (44.8%)	χ^2^ = 9.764, *p* = 0.002

AHI—apnea-hypopnea index, AI—apnea index, HI—hypopnea index, ODI—oxygen desaturation index, OAH—obstructive apnea-hypopnea index, CAH—central apnea-hypopnea index, MAH—mixed apnea-hypopnea index, SDB—sleep-disordered breathing.

**Table 6:** Factors associated with poor outcome (based on Cox proportional hazard regression models).

	Model 1 (Incl. Hypoxemia Time < 2.1% versus ≥2.1% Nocturnal Time)		Model 2 (Incl. Average Nocturnal Desaturation Drop < 3.65% versus ≥3.65%)	
Parameter	HR (95% CI)	*p*-Level	HR (95% CI)	*p*-Level
Age	1.091 (1.040; 1.140)	<0.001	1.062 (1.020; 1.107)	0.004
Sex (male)	excl	0.985	excl	0.368
TOAST	1.339 (0.974; 1.839)	0.072	1.455 (1.065; 1.989)	0.019
Atrial fibrillation (yes)	5.075 (1.961; 13.137)	0.001	4.231 (1.666; 10.741)	0.002
Thrombolytic therapy (yes)	excl	0.447	Excl	0.330
Emergency vascular intervention (yes)	excl	0.708	Excl	0.724
Previous stroke/TIA	2.100 (0.897; 4.916)	0.087	Excl	0.114
Obesity (yes)	excl	0.249	Excl	0.279
Diabetes mellitus (yes)	2.303 (1.037; 5.118)	0.041	excl	0.305
Coronary artery disease (yes)	2.261 (0.950; 5.381)	0.065	excl	0.137
Hypertensive crisis at stroke onset (yes)	excl	0.332	excl	0.463
NIHSS at discharge	1.158 (1.071; 1.252)	<0.001	1.106 (1.026; 1.191)	0.008
eGFR	excl	0.569	excl	0.221
Triglycerides	0.456 (0.218; 0.953)	0.037	0.515 (0.256; 1.036)	0.063
C-reactive protein	1.019 (1.005; 1.032)	0.007	1.024 (1.010; 1.038)	0.001
Platelet count	1.009 (1.004; 1.015)	0.001	1.009 (1.004; 1.015)	0.001
Respiratory rate ≥ 15.6/min	excl	0.268	excl	0.921
SpO_2_ < 90% during ≥2.1% of total analyzed time	3.693 (1.517; 8.992)	0.004	-	-
Average nocturnal desaturation drop ≥ 3.65%	-	-	4.257 (1.612; 11.240)	0.003

Cox proportional hazard regression models with stepwise exclusion. Excl—factor excluded from the model.

## Data Availability

The datasets generated during and/or analyzed during the current study are available from the corresponding author on reasonable request and with permission from Almazov National Medical Research Centre.

## Supplementary Materials

The following supporting information can be downloaded at: https://www.mdpi.com/article/10.3390/diagnostics13132246/s1, Figure S1: Survival (Kaplan–Meier curves) depending on the hypoxemia burden in patients with previous stroke; Table S1: Characteristics of the patients with previous stroke; Table S2: Factors associated with poor outcome (based on Cox proportional hazard regression models).
