# Supplementary material for: Sleep-Disordered Breathing and Prognosis after Ischemic Stroke: It Is Not Apnea-Hypopnea Index That Matters
Source: Diagnostics (Basel). 2023 Jul 3;13(13):2246. doi: 10.3390/diagnostics13132246 (PMC10340264; doi:10.3390/diagnostics13132246)
Supplement: Supplementary file 1 [file diagnostics-13-02246-s001.zip › diagnostics-2413812-supplementary.pdf]

diagnostics

0Supplementary Materials: Sleep-disordered breathing and prognosis after ischemic stroke: it is not apnea-hypopnea index that matters

Lyudmila Korostovtseva [1,*], Mikhail Bochkarev [1], Valeria Amelina [1,2], Uliana Nikishkina [3], Sofia Osipenko [1,3], Anastasia Vasilieva [1], Vladislav Zheleznyakov [1], Ekaterina Zabroda [1,4], Alexey Gordeev [1,4], Maria Golovkova-Kucheryavaia [1], Stanislav Yanishevskiy [1], Yurii Sviryaev [1] and Aleksandra Konradi [1]

A subanalysis of the subgroup with previous stroke.

The patients with previous stroke did not differ from the whole cohort by sex (p=0.89), age (p=0.89), stroke subtype (p=0.96), cerebral artery involvement (p=0.06), NIHSS at admission (p=0.23) and at discharge (p=0.087), as well as by nocturnal respiratory parameters (p>0.05 for all measures). At the same time, thrombolytic therapy was administered less frequently in patients with previous stroke compared to those experiencing their first-ever stroke: 5 (5.4%) versus 26 (13.9%), χ^2=4.587, p=0.022. With regard to comorbidities, almost all patients with previous stroke were hypertensive: 91 (97.8%) versus 171 (91.4%) (χ^2=4.237, p=0.040). Other comorbidities were registered with equal frequency in patients with and without previous stroke.

Table S1. Characteristics of the patients with previous stroke.

Parameter	Total (n=93)	Patients with poor survival (n=21)	Patients free of events (n=72)	p-level
**Clinical and demographic parameters**				
Sex (m), n (%)	54 (58.1%)	14 (66.7%)	40 (55.6%)	χ^2=0.824, p=0.36
Age, years	66.2±10.2	69.9±10.9	65.1±9.8	p=0.048
HTN, n (%)	91 (97.8%)	21 (100%)	70 (97.2%)	χ^2=0.596, p=0.44
HTN crisis, n (%)	35 (10.7%)	5 (7.7%)	30 (11.4%)	χ^2=1.667, p=0.20
AF, n (%)	27 (29%)	9 (42.9%)	18 (25%)	χ^2=2.516, p=0.11
CAD, n (%)	50 (53.8%)	14 (66.7%)	36 (50%)	χ^2=1.817, p=0.18
Previous MI, n (%)	21 (22.6%)	8 (38.1%)	13 (18.1%)	χ^2=3.735, p=0.053
Diabetes mellitus, n (%)	28 (30.1%)	7 (33.3%)	21 (29.2%)	χ^2=0.134, p=0.71
Obesity, n (%)	23 (24.7%)	6 (28.6%)	17 (23.6%)	χ^2=0.215, p=0.64
**Stroke characteristics**				
**TOAST classification** (p=0.56)				
Atherothrombotic	15 (16.1%)	3 (14.30%)	12 (16.7%)	
Cardioembolic	30 (32.3%)	7 (33.3%)	23 (31.9%)	
Small-vessel disease	12 (12.9%)	1 (4.8%)	11 (15.3%)	
Other determined etiology	1 (1.1%)	0	1 (1.4%)	
Undetermined etiology	35 (37.6%)	10 (47.6%)	25 (34.7%)	
**Affected cerebral artery** (p=0.88)				
Vertebrobasilar (posterior cerebral artery)	16 (17.2%)	4 (19.1%)	12 (16.7%)	
Anterior cerebral artery	1 (1.1%)	0	1 (1.1%)	
Median cerebral artery	68 (73.1%)	15 (71.4%)	53 (73.6%)	
Several arteries	8 (8.6%)	2 (9.5%)	6 (8.3%)	
**Medical interventions**				
Thrombolytic therapy, n	5 (5.4%)	1 (4.8%)	4 (5.6%)	χ^2=0.020, p=0.89

(%)				
Emergency stroke-related vascular surgery	18 (19.4%)	5 (23.8%)	13 (18.1%)	χ^2=0.345, p=0.56
NIHSS baseline, score	4 (1; 25)	6 (1; 25)	4 (1; 25)	0.031
NIHSS baseline≥5 scores, n (%)	53 (57%)	15 (71.4%)	38 (52.8%)	χ^2=2.96, p=0.085
NIHSS discharge, score	3 (0; 23)	3(0; 23)	3 (0; 19)	0.34
**Respiratory parameters**				
AHI, episodes/h	13.9 (0.0; 86.5)	11.2 (0.6; 54.1)	15.9 (0.0; 86.5)	0.51
ODI, episodes/h	10.5 (0.0; 79.3)	7.4 (1.2; 45.1)	13.7 (0.0; 79.3)	0.083
Respiratory rate at night, breaths/min	15.9 (11.2; 29.0)	16.6 (11.4; 27.8)	15.7 (11.2; 29.0)	0.78
Average SpO_2, %	92.7 (84.6; 97.4)	92.6 (86.4; 96.8)	92.8 (84.6; 97.4)	0.77
Average desaturation drop, %	3.85 (1.3; 9.4)	4.1 (3.2; 7.1)	3.8 (2.5; 9.3)	0.11
SpO_2 <90%, percent of total analyzed time	5.1 (0.0; 87.9)	6.2 (0.0; 31)	2.0 (0.0; 87.9)	0.29
SDB (AHI≥5/h), n (%)	67 (72%)	13 (61.9%)	54 (75.0%)	χ^2=1.384, p=0.24
Hypoxemia (SpO_2<90%) ≥2.1% of total analyzed time, n (%)	49 (52.6%)	15 (53.6%)	35 (47.2%)	
Average desaturation drop ≥3.65%, n (%)	53 (57%)	14 (66.6%)	39 (54.2%)	χ^2=2.511, p=0.11

The survival rate was poorer in patients with a higher nocturnal hypoxemia burden (SpO_2<90 % during ≥2.1% of total analyzed time)—29.2 (21.9; 36.4) months compared to 38.0 (33.8; 42.1) months in those with a lower nocturnal hypoxemia burden (SpO_2<90 % during <2.1% of total analyzed time) (Log Rank 5.366, p=0.021) (Fig. 1S).

No difference was found between groups with higher (≥3.65%) versus lower (<3.65%) desaturation drops during night: 40.4 (34.1; 46.7) versus 32.2 (25.6; 38.8) months (Log Rank 1.859, p=0.173).

The survival rate did not differ depending on the presence of SDB (AHI<5 versus AHI≥5/h): 23.5 (16.1; 30.8) versus 36.2 (30.7; 41.7) months (Log Rank 2.840, p=0.092).

In a multivariable Cox proportional hazards regression (backward stepwise analysis) model, only age was associated with survival time, while the parameters of hypoxemia burden had borderline significance (Table 2 Suppl).

Table S2. Factors associated with poor outcome (based on Cox proportional hazard regression models).

	**Model (incl. hypoxemia time <2.1% versus ≥2.1% nocturnal time)**	
**Parameter**	**HR (95%CI)**	**p-level**
Age	1.082 (1.020; 1.147)	0.008
Sex (male)	excl	0.234
Thrombolytic therapy (yes)	excl	0.598
Emergency vascular intervention (yes)	excl	0.803
Coronary artery disease (yes)	excl	0.613
Diabetes mellitus (yes)	excl	0.237
NIHSS at admission	excl	0.085
SpO_2<90% during ≥2.1% of total analyzed time	3.360 (0.930; 12.146)	0.065

Cox proportional hazard regression models with stepwise exclusion. Excl—factor excluded from the model.

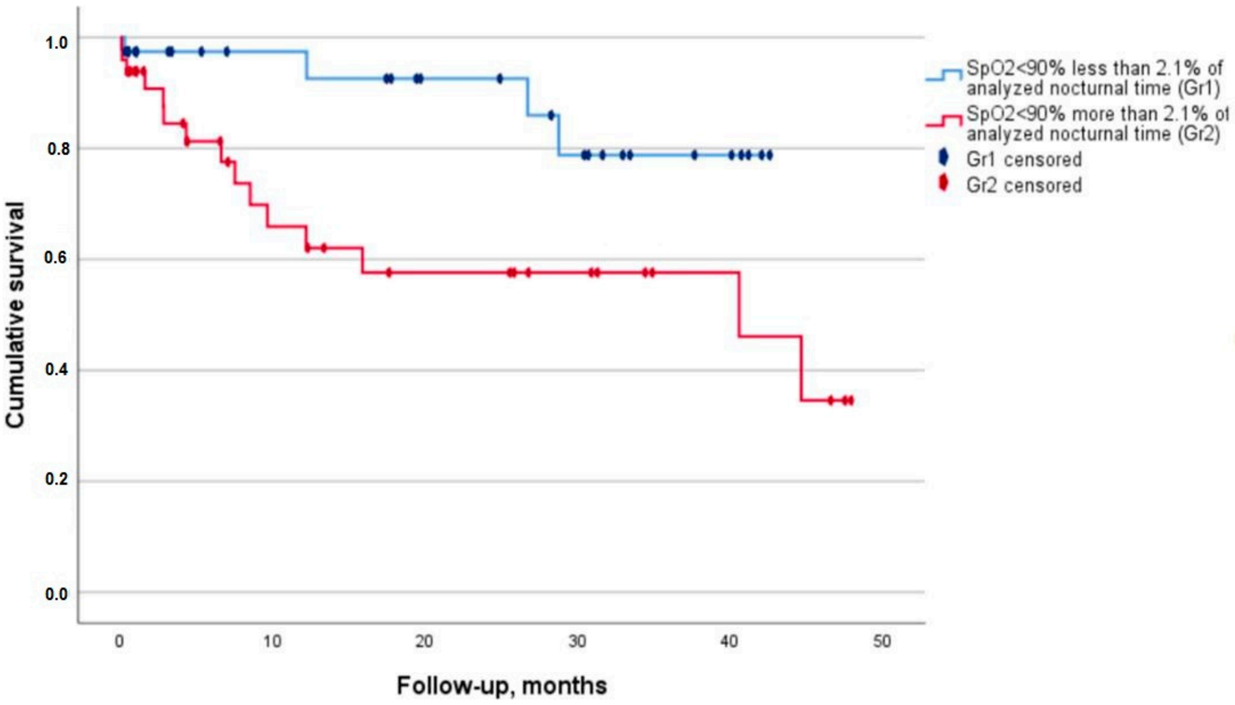

Figure S1. Survival (Kaplan–Meier curves) depending on the hypoxemia burden in patients with previous stroke.
